# RNA Sequencing and Metabolomic Analyses Reveal Differences in Muscle Characteristics and Metabolic Profiles Between Purebred and Crossbred Huainan Pigs

**DOI:** 10.3390/ani15213144

**Published:** 2025-10-29

**Authors:** Jing Wang, Yufu Li, Mengyang Zhang, Junfeng Chen, Qingxia Lu, Hanbing Zhang, Xiangzhou Yan, Chuanying Pan, Xuelian Zhang, Baosong Xing

**Affiliations:** 1 Henan Key Laboratory of Farm Animal Breeding and Nutritional Regulation, Henan Pig Breeding Engineering Research Centre, Institute of Animal Husbandry, Henan Academy of Agricultural Sciences, Number 116, Hua Yuan Road, Zhengzhou 450002, China; 2 Jiangsu Key Laboratory of Sericultural Biology and Biotechnology, School of Biotechnology, Jiangsu University of Science and Technology, Zhenjiang 212000, China; 3 Key Laboratory of Animal Genetics, Breeding and Reproduction of Shaanxi Province, Key Laboratory of Animal Biotechnology, College of Animal Science and Technology, Northwest A&F University, Ministry of Agriculture, Number 22, Xi Nong Road, Yangling 712100, China

**Keywords:** Huainan pig, hybridization, muscle quality, RNA-seq, metabolomics

## Abstract

**Simple Summary:**

This study aimed to enhance the low lean meat yield and slow growth rate of Huainan (HN) pigs through crossbreeding, resulting in three hybrid combinations (BH, LH, and YH) by mating HN pigs with Berkshire, Landrace, and Yorkshire pigs, respectively. Offspring were assessed for muscle characteristics, and the longissimus dorsi (LD) muscle underwent extensive transcriptomic and metabolomic evaluations. Evaluation of meat quality assessment indicated that all three crossbreeding strategies increased the proportion of lean meat in HN pigs. Integrated transcriptomic and metabolomic profiling disclosed significant disparities among the genetic groups and identified crucial differentially expressed genes (DEGs) and differentially accumulated metabolites (DAMs). These findings offer valuable insights for improving the meat quality of Huainan pigs and elevating the standard of derived products.

**Abstract:**

The HN pig, indigenous to Henan Province, is distinguished by its reduced lean meat yield and slower growth rates relative to commercial foreign breeds. To address these limitations, three hybrid combinations were generated through the crossbreeding of Huainan sows with Yorkshire, Landrace, and Berkshire sires. In this study, extensive transcriptomic and metabolomic analyses of the LD muscle were carried out for the first time, and carcass and meat quality characteristics were compared between hybrid and HN pigs. Slaughter and muscle quality assessments revealed that the lean meat percentage of LH and YH was significantly lower than that of HN, with YH exhibiting the lowest intramuscular fat level, indicating that this breed possesses enhanced lean meat production efficiency. Transcriptomic profiling revealed markedly increased expression of *SLIT2*, *CH25H*, *NR4A2*, *NR4A1*, *FOSB*, *CRABP2*, *GDF10*, and *MRAP2* in all three hybrid groups compared to HN. Gene Ontology enrichment analysis identified that the skeletal muscle cell differentiation (GO:0035914) and transforming growth factor beta receptor signaling pathway (GO:0007179) were exclusively enriched in the YH vs. HN comparison. Non-targeted metabolomic analysis identified 31, 36, and 12 DAMs in BH vs. HN, LH vs. HN, and YH vs. HN comparisons, with pyruvate metabolism being the sole pathway common to all groups. An integrated multi-omics analysis revealed significant correlations between phytosphingosine levels and DEGs across all three comparisons. In summary, these results indicate that crossbreeding substantially improves lean meat yield in HN pigs while providing novel molecular insights into the underlying genetic and metabolic mechanisms.

## 1. Introduction

Prior to the 20th century, pig breeding in China primarily relied on traditional self-breeding and small-scale household rearing, leading to the development of numerous indigenous pig breeds with distinct regional characteristics [1]. The high intramuscular fat (IMF) content of the Huainan pig, one of China’s prized native breeds, can enhance the flavor of the meat; nevertheless, the advantages of IMF vary by animal and geographical location. Additionally, the breed has a high litter size, a good capacity to use roughage efficiently, and a tolerance for heat [2]. It is generally believed that increased IMF levels have a positive effect on the sensory quality of pork [3]. Studies have shown that the genetic selection to reduce subcutaneous fat significantly reduced IMF in commercial hybrid pigs (2.5%), which had a strong negative impact on the sensory characteristics of pork (flavor, juiciness, and tenderness) and consumer acceptance. Despite their distinct benefits in terms of meat quality, Huainan pigs are less suited to contemporary intensive production systems than commercial pig breeds due to their low lean meat percentage, moderate development rate, and prolonged feeding cycle. Commercially introduced varieties like Yorkshire, Berkshire, and Duroc, on the other hand, grow quickly and produce a lot of lean meat, but their meat is of lower quality and flavor [4,5,6]. With the increasing demand of consumers for both pork quality and breeding efficiency, how to improve the growth rate and lean meat yield of Huainan pigs on the premise of ensuring excellent meat quality has become an urgent problem to be solved. Therefore, crossbreeding Huainan pigs with foreign commercial breeds has become an important strategy to improve lean meat yield and growth performance while preserving superior meat quality.

In genetic breeding research, the phenotypic analysis of single traits is inadequate to clarify the regulatory mechanisms underlying complex economic traits such as IMF deposition [7]. The integration of multi-omics technologies, particularly the combined analysis of the transcriptome and metabolome, provides a powerful approach to systematically reveal the associations between gene expression and metabolic changes [8]. The integration of transcriptomic and metabolomic methodologies enhances comprehension of the impact of genes on essential meat quality characteristics, including fat deposition, via the modulation of metabolic pathways at various levels. Yu et al. conducted transcriptome, metabolomic, and proteomic investigations on Qinling Black pigs (QLB) and Large White pigs (LW), identifying *RapGEF1* as a key gene regulating IMF content [9]. Dan et al. crossbred the Chinese obese pig breed Neijiang (NJ) with the lean Large White (LW) to develop the Neijiang × Large White (NL) hybrid. They conducted a comparative analysis of carcass features and meat quality parameters of the two breeds utilizing transcriptome and metabolomic methodologies, therefore validating the potential for enhancing the production performance of Neijiang pigs by hybridization with Large White [10]. The molecular mechanisms underlying fat deposition and the enhancement of meat quality in Huainan pigs through different hybridization strategies remain unclear.

This study thoroughly analyzed the impact of various hybridization procedures on meat quality enhancement in Huainan pigs by utilizing Huainan pigs as the maternal line and crossbreeding them with Berkshire, Landrace, and Yorkshire pigs to produce three hybrid combinations: BH, LH, and YH. Purebred Huainan pigs (HN) served as the control group to evaluate differences in carcass traits and meat quality between the hybrids and the native breed. Variations in paternal genetic backgrounds may activate different biochemical pathways, influencing muscle growth and fat deposition in the progeny at several levels [11]. The LD muscle is frequently utilized as a representative tissue for examining carcass composition and meat quality characteristics in swine. The LD muscle represents overall carcass composition and is strongly associated with important pork quality traits [12]. Consequently, LD muscle tissues were collected from four groups to examine metabolic differences between hybrid pigs and Huainan pigs, and to identify potential regulatory factors via an integrated transcriptomic and metabolomic analysis. This study seeks to offer key data and insights into the molecular mechanisms by which various hybrid combinations modulate meat quality. The findings will provide a scientific foundation for molecular breeding, enhancement of meat quality, and optimization of hybridization strategies in Huainan pigs.

## 2. Materials and Methods

### 2.1. Ethics Statement

The experimental protocols in this study were approved by the Institutional Animal Care and Use Committee of Henan Academy of Agricultural Sciences (1 August 2023) [13]. The care and use of animals in this experiment strictly adhere to local policy standards and animal welfare legislation.

### 2.2. Sample Collection

**Figure 1:**
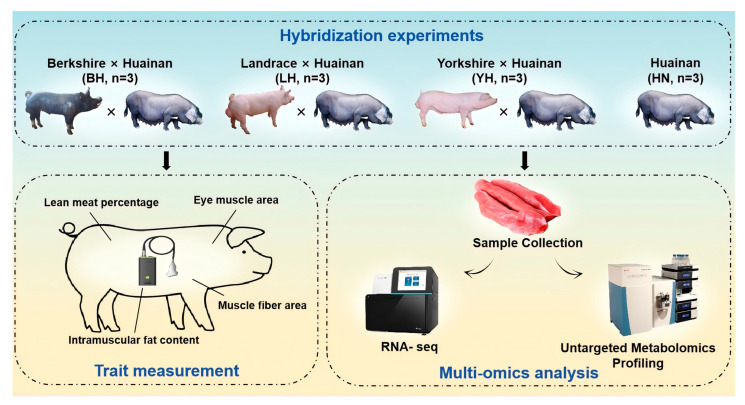
The overall experiments and analysis.

The experiment was conducted at the Huainan Pig Farm operated by Henan Xingrui Agriculture and Animal Husbandry Technology Co., Ltd. Sixty Huainan sows, in their second to third parity and of similar age and body condition, were selected and randomly divided into three groups, with 20 sows in each group. The sows were inseminated with semen collected from four boars of the same paternal line within each of the three breeds (Yorkshire, Landrace, or Berkshire), resulting in three hybrid combinations: Yorkshire × Huainan (YH), Landrace × Huainan (LH), and Berkshire × Huainan (BH). Ten castrated male pigs with a body weight of approximately 30 kg were selected from each of the three hybrid progeny groups and the Huainan pig group (HN) for a fattening trial (Figure 1). All pigs were raised in identical conditions, with unrestricted access to feed and water. The experimental feed formula was consistent with previous experiments, comprising 58% corn, 14% soybean meal, 9% wheat bran, 15% grass meal, and 4% premix (Table 1) [14]. The prescribed nutrition standards (NY/T 65-2004) were met by other nutrients [15]. Antibiotics and any other medications were not administered to the sows and offspring during the experiment.

### 2.3. Carcass and Muscle Characteristics Measurements

When the pigs weighed around 100 kg, three pigs with similar weights and body conditions were randomly selected from each group for slaughter by electrical stunning. Prior to slaughter, pigs were deprived of food for 24 h, with free access to water, and exhibited no aggressive behavior. Body weight was measured two hours before slaughter. Following the exsanguination of each pig, the hair was removed, and the head, hooves, tail, and internal organs were discarded, while the kidney and plate oil were retained. The carcass was bisected along the midline, and the weights of both halves were summed. The loin eye area (height × width × 0.7, cm^2^) and average backfat thickness (mm) at the shoulder, last rib, and loin were measured using a vernier caliper [16]. Subsequently, the left side of the carcass was dissected to separate all the lean meat, bones, fat, and skin. The dressing percentage was calculated as the percentage of carcass weight to slaughter body weight, while the lean-to-fat ratio was calculated as the proportion of lean meat to carcass weight [10]. Detailed records were made of their carcass and muscle characteristics, including eye muscle area, lean meat percentage, IMF content, and muscle fiber area. For subsequent analysis, the LD tissue from the left side of the carcass at the 6th to 7th rib was obtained, and three biological replicate samples were taken from each group. The collected samples were preserved in liquid nitrogen for future investigations.

### 2.4. IMF and Moisture

IMF content was determined using the Soxhlet method, while moisture content was measured using the atmospheric heating drying method [17]. Briefly, connective tissues at the edges of the LD were trimmed, and the remaining tissue was pulverized. Approximately 15 g of minced meat was evenly spread in a pre-weighed tray and dried in a hot-air oven at 102 °C until a constant weight was achieved. Each sample was measured in duplicate. For IMF determination, the dried meat was powdered, and ~1.8 g of meat powder was used for crude fat determination with a Soxhlet extractor. Each sample was measured in triplicate. The mean values of moisture and crude fat contents were calculated and used as the final results. IMF and moisture contents from the anterior and posterior portions of the LD were averaged for each animal, as no significant differences were observed between the two portions.

### 2.5. RNA Extraction, Library Construction, and Sequencing

Total RNA was extracted utilizing Trizol reagent (Thermo Fisher, 15596018, Carlsbad, CA, USA), and its quantity and purity were evaluated with the Bioanalyzer 2100 and RNA 6000 Nano LabChip Kit (Agilent, 5067-1511, Santa Clara, CA, USA). RNA samples of superior quality (RIN > 7.0) were selected for library construction. mRNA was purified from 5 μg of total RNA utilizing Dynabeads Oligo(dT) (Thermo Fisher) and subsequently reverse-transcribed into cDNA with SuperScript™ II Reverse Transcriptase (Invitrogen, Thermo Fisher Scientific, 1896649, Carlsbad, CA, USA). U-labeled DNA underwent treatment with UDG enzyme (NEB, m0280, Ipswich, MA, USA), followed by PCR amplification of the ligated products. Ultimately, 2 × 150 bp paired-end sequencing (PE150) was performed on an Illumina Novaseq™ 6000 (LC-Bio Technology, Hangzhou, China).

### 2.6. Quantitative Real-Time PCR (qRT-PCR) Validation

Primers were designed using Primer-BLAST (https://www.ncbi.nlm.nih.gov/tools/primer-blast, accessed on 2 September 2024), with the primer sequences enumerated in Table S1. The primers were produced by Bioengineering Co., Ltd. (Shanghai, China). Total RNA was extracted from the LD muscle of Huainan pigs and three hybrid offspring groups, followed by cDNA synthesis utilizing the Hifair^®^ III 1st Strand cDNA Synthesis Super Mix for qPCR kit (Yeasen Biotechnology, Shanghai, China). Subsequently, real-time qPCR analysis was conducted utilizing the ChamQ SYBR qPCR Master Mix kit (Vazyme Biotech, Nanjing, China). The reaction system (13 µL) consisted of 6.5 µL of 2 × ChamQ SYBR qPCR Master Mix, 0.5 µL of each upstream and downstream primer, 5 µL of cDNA diluted 40 times, and 0.5 µL of double-distilled water (ddH_2_O). The qRT-PCR reaction conditions were as follows: initial denaturation at 95 °C for 30 s, followed by 40 cycles of denaturation at 95 °C for 10 s and annealing at 60 °C for 30 s, culminating in a melting curve acquisition protocol. The relative gene expression levels were calculated using the 2^−∆∆Ct^ method.

### 2.7. Analysis of DEGs and Pathways

Differential expression analysis was performed using DESeq2 (v1.46.0) [18] for group comparisons and edgeR for individual samples. DEGs were identified with an FDR < 0.05 and an absolute fold change ≥ 2. Gene Ontology (GO) enrichment analysis of DEGs was conducted with the goseq R package (version 1.60.0), adjusting for gene length bias. Kyoto Encyclopedia of Genes and Genomes (KEGG) pathway enrichment analysis was performed via KOBAS (v3.0) [19], with a corrected *p*-value threshold of <0.05 considered significant.

### 2.8. Untargeted Metabolomics Profiling

Untargeted metabolomics services were provided by Lc-Bio Technologies (Hangzhou, China) Co., Ltd. The samples, which were stored at −80 °C in a freezer, were thawed on ice. After acquiring a 50 mg sample, homogenization and extraction were performed. After centrifugation, 200 µL aliquots of the supernatant were transferred for LC-MS analysis. Chromatographic separation was performed using an ACQUITY UPLC HSS T3 column (ACQUITY Premier HSS T3, 1.8 µm, 2.1 × 100 mm; Waters, Milford, MA, UK). The column temperature was sustained at 40 °C, with a flow rate of 0.35 mL/min [20]. The mobile phase comprised solvent A (water with 5 mmol/L ammonium acetate and 5 mmol/L acetic acid) and solvent B (LC–MS grade acetonitrile) [21]. Data acquisition was conducted using a high-resolution Q Exactive mass spectrometer (Thermo Fisher Scientific, Bremen, Germany) [22]. Each sample was evaluated once in positive ion mode and once in negative ion mode. Quality control (QC) samples were inserted evenly across each group or batch to assess instrument stability and correct systemic errors.

### 2.9. Mass Spectrometry Data Analysis

The acquired mass spectrometry data were preprocessed using XCMS software (version 3.20) for peak detection, peak alignment, retention time correction, peak grouping, and annotation of isotopes and adducts. The raw LC–MS data files were first converted to the mzXML format, followed by processing with XCMS and the metaX toolbox (version 1.4) in R. Ion features were identified by combining retention time (RT) and *m*/*z* information. The intensity of each peak was recorded, and a three-dimensional data matrix was generated, consisting of peak indices (RT–*m*/*z* pairs), sample identifiers (observations), and ion intensity values (variables).

### 2.10. Statistical Analysis

When analyzing carcass traits and muscle characteristics, one-way analysis of variance (ANOVA) was used for statistical comparisons. Prior to ANOVA, the data were tested for normality using the Shapiro–Wilk test and for homogeneity of variances using Levene’s test, and all datasets met these assumptions. Statistical analyses were performed using SPSS software (SPSS, Inc., Chicago, IL, USA, version 27). An ANOVA was used to examine the differences among treatments, and when significant differences were identified, a Tukey test was employed to assess those differences [23]. A *p* < 0.05 (two-tailed) was considered statistically significant. Data were expressed as mean ± SEM and were derived from at least three independent experiments. The data visualization for qRT-PCR was executed utilizing GraphPad Prism (version 10.1.2) and Origin 2024 (version 10.1) software.

## 3. Results

### 3.1. Evaluation of Carcass and Muscle Characteristics

We compared data on eye muscle area, lean meat percentage, IMF content, and muscle fiber area among three hybrid groups (YH, BH, and LH) as well as the Huainan pig group (HN) (Table 2). The results showed that the eye muscle area was significantly greater in the BH, LH, and YH groups than in the HN group, with the YH group exhibiting the largest value. The lean meat percentage in the YH and LH groups was significantly higher than that of the HN group, with the YH group exhibiting the greatest proportion. The HN group had significantly higher IMF content than the YH group, whereas among the three hybrid groups, the LH group possessed the greatest level. The HN group exhibited markedly larger muscle fibers compared to the YH group, which also demonstrated significantly larger fibers than the LH group. Among the crossbred groups, YH pigs showed the greatest eye muscle area, lean meat percentage, and muscle fiber area, but the least IMF content, whereas LH pigs exhibited the smallest muscle fiber area and the highest IMF content. These findings indicate that different hybrid groups may possess distinct advantages.

### 3.2. Analysis of DEGs in Each Hybrid Group Compared to the Purebred Group

**Figure 2:**
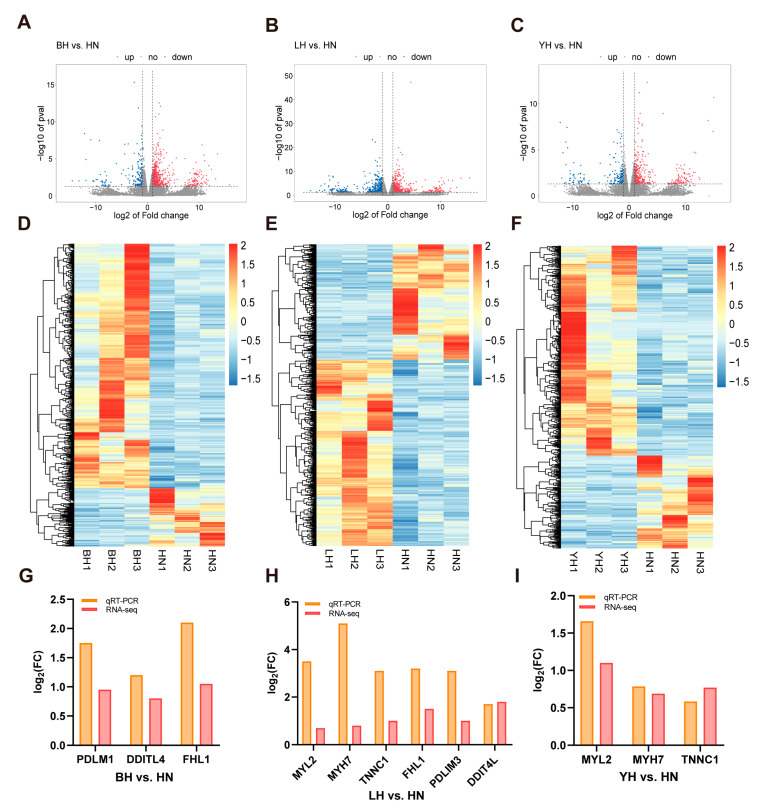
Transcriptomic analysis and quantitative real-time PCR validation of the LD muscle in the three hybrid pig groups compared with the purebred Huainan pigs. Volcano plots of DEGs: BH vs. HN (**A**), LH vs. HN (**B**), YH vs. HN (**C**); clustering heatmaps of DEGs: BH vs. HN (**D**), LH vs. HN (**E**), YH vs. HN (**F**); fold changes in expression levels of DEGs: BH vs. HN (**G**), LH vs. HN (**H**), YH vs. HN (**I**).

To further investigate the effects of various hybrid combinations on LD muscle development and IMF deposition capacity, we performed transcriptome sequencing on LD muscle tissues from four groups of pigs. Twelve LD samples were subjected to transcriptome sequencing, resulting in a range of 34,690,370 to 44,344,944 raw reads. After data filtering, the quantity of clean reads varied between 33,479,402 and 42,817,002, with valid reads constituting over 96.51% of the total. The Q20 and Q30 values exceeded 99.73% and 97.43%, respectively, while the mean GC content across all samples was 52.08% (Table S2). DEGs in the BH, LH, YH, and HN groups were analyzed using DESeq2 (Table S3). A total of 743, 777, and 433 DEGs were identified in the comparisons of BH vs. HN, LH vs. HN, and YH vs. HN, respectively. Specifically, in comparison to HN, 631 genes were up-regulated and 112 down-regulated in BH; 544 genes were up-regulated and 233 down-regulated in LH; and 323 genes were up-regulated and 110 down-regulated in YH (Figure 2A–C). The heatmap illustrates the expression profiles of all DEGs across the three comparison groups, suggesting potential biological disparities (Figure 2D–F).

We focused on DEGs that were significantly enriched across all three pairwise comparisons. The results revealed that in all hybrid groups, genes including *SLIT2*, *CH25H*, *NR4A2*, *NR4A1*, *FOSB*, *CRABP2*, *GDF10*, and *MRAP2* were expressed at markedly elevated levels relative to the HN group. The expression levels of *BTG2*, *EGR3*, *CCN1*, and *FKBP5* were significantly elevated in the HN group compared to the three hybrid groups. Six genes were randomly selected from the list of functional DEGs for validation, and their expression levels were confirmed to differ significantly by RT-PCR (Figure 2G–I). The expression trends of these genes were entirely congruent with the RNA-seq results.

### 3.3. GO and KEGG Enrichment Analysis

**Figure 3:**
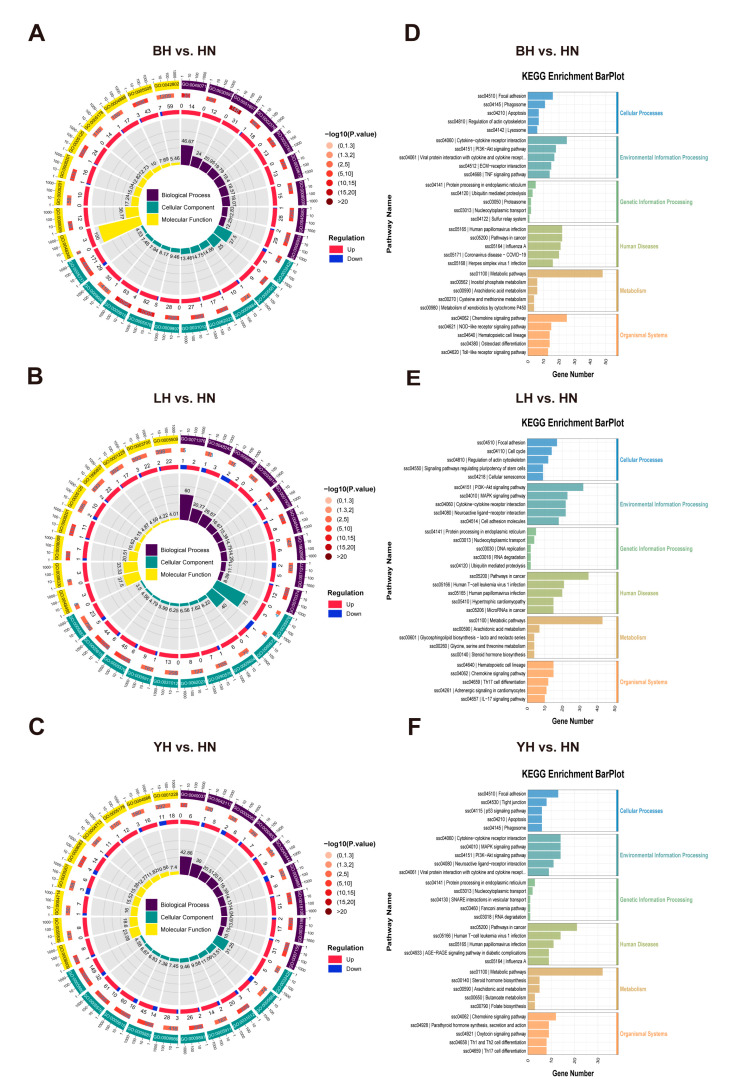
GO and KEGG enrichment analyses of DEGs in BH vs. HN (**A**,**D**), LH vs. HN (**B**,**E**), YH vs. HN (**C**,**F**).

To examine the molecular differences resulting from hybridization, we performed enrichment analysis of the identified DEGs (*p* < 0.05) utilizing the GO and KEGG databases. The findings indicated that these DEGs were primarily abundant in biological processes and pathways related to adipocyte development, immune response, inflammatory response, cell adhesion, and signal transduction. In the comparison between BH and HN, DEGs were categorized into 55 GO subcategories, including 29 terms related to biological processes, 11 phrases pertaining to cellular components, and 15 terms associated with molecular functions. In the comparison between LH and HN, 64 GO subcategories were identified, including 39 biological process terms, 12 cellular component terms, and 13 molecular function terms. 37 subcategories were annotated for the YH vs. HN comparison, including terminology for 4 cellular components, 11 chemical functions, and 22 biological processes. Figure 3A–C illustrates the top 20 enriched pathways for each group. GO enrichment analysis revealed that fat cell differentiation (GO:0045444) was significantly enriched across all three comparison groups, implying that hybridization influences adipocyte differentiation (Tables S4–S6). Skeletal muscle cell differentiation (GO:0035914) and the transforming growth factor beta receptor signaling pathway (GO:0007179) were exclusively enriched in the YH vs. HN group (Table S6).

KEGG pathway analysis demonstrated substantial enrichment of several signaling pathways in the BH vs. HN comparison, with the top 30 pathways illustrated in Figure 3D. Several immune-related pathways were discovered, including the TGF-β signaling pathway, NOD-like receptor signaling pathway, Toll-like receptor signaling pathway, and RIG-I-like receptor signaling pathway (Figure 3D, Table S7). Likewise, KEGG analysis of the LH vs. HN comparison revealed that significant pathways, including PI3K-Akt, MAPK, FoxO, and Rap1 signaling pathways, were enriched with more genes (Figure 3E, Table S8). In contrast, the comparison between YH and HN comparison had the fewest enriched pathways, predominantly featuring genes involved in cytokine–cytokine receptor interactions and the chemokine signaling pathway, primarily linked to the immune system and signaling molecule interactions (Figure 3F, Table S9). These findings suggest that the enriched pathways and associated biological processes may contribute to the observed differences in muscle characteristics between hybrid offspring and Huainan pigs.

### 3.4. DAMs and Metabolome Data Analysis

**Figure 4:**
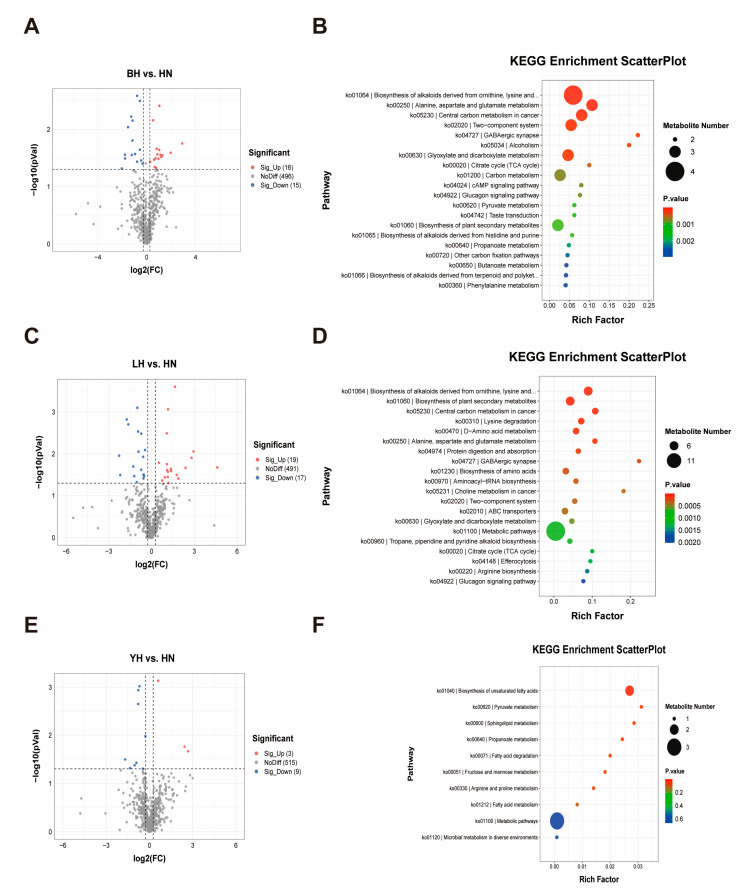
Analyses of DAMs. Volcano plot of DAMs between BH and HN (**A**) and KEGG enrichment analysis (**B**); volcano plot of DAMs between LH and HN (**C**) and KEGG enrichment analysis (**D**); volcano plot of DAMs between YH and HN (**E**) and KEGG enrichment analysis (**F**).

We employed untargeted metabolomics profiling to investigate the mechanism underlying the disparity in muscle quality by identifying DAMs in the LD muscle between hybrid offspring and Huainan pigs. A clear distinction between the hybrid offspring and Huainan pigs was shown by clustering analysis according to the DAMs. A total of 527 metabolites were detected among the four comparisons (Table S10). The primary metabolites included lipids and lipid-like molecules (25.04%), organic benzenoid compounds (20.31%), organic acids and their derivatives (19.17%), benzenoid organics (9.87%), and acids along with their derivatives (9.30%). With VIP ≥ 1 and *p* < 0.05 as screening criteria, the DAMs were found in the four comparisons. A total of 31 DAMs were identified in the BH vs. HN comparison, with 16 exhibiting up-regulation and 15 down-regulation (Figure 4A). In the LH vs. HN comparisons, 36 DAMs were found, comprising 19 that were up-regulated and 17 that were down-regulated (Figure 4C). In the comparison between YH vs. HN, 12 DAMs were found, comprising 3 that were up-regulated and 9 that were down-regulated (Figure 4E).

According to KEGG pathway enrichment analysis, the DAMs in the comparison of BH and HN were enriched in 66 pathways, encompassing critical lipid metabolism pathways such as glycerophospholipid metabolism (ko00564), regulation of adipocyte lipolysis (ko04923), and pyruvate metabolism (ko00620) (Figure 4B, Table S11). DAMs of LH and HN were enriched in a total of 41 pathways (Figure 4D, Table S12). Similar to the BH vs. HN comparison, pathways related to lipid metabolism, including glycerophospholipid metabolism and pyruvate metabolism, were likewise enriched in this group. The primary regulatory pathway, mTOR signaling (ko04150), was highly enriched. The comparison between YH and HN revealed 12 DAMs concentrated throughout 6 pathways, predominantly in the production of unsaturated fatty acids (ko01040) and pyruvate metabolism (Figure 4F, Table S13).

### 3.5. Combined Analysis of Transcriptome and Metabolomics

**Figure 5:**
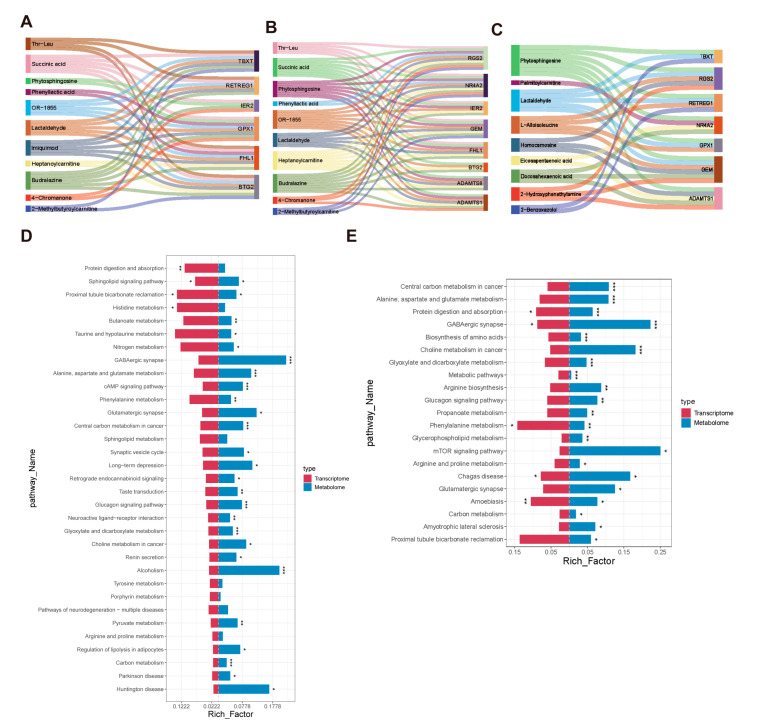
Correlation networks and common metabolic pathways between DAMs and DEGs in LD muscle. Panels (**A**–**C**): Correlation networks between DAMs and DEGs for BH vs. HN (**A**), LH vs. HN (**B**), and YH vs. HN (**C**), respectively. Panels (**D**,**E**): Common metabolic pathways annotated by DAMs and DEGs for BH vs. HN (**D**) and LH vs. HN (**E**). *, **, and *** denote statistical significance at *p* < 0.05, *p* < 0.01, and *p* < 0.001, respectively.

Pearson’s correlation coefficient was used to analyze the correlation between DAMs and DEGs, and the coefficients were calculated from the metabolite areas and FPKM values of the DEGs. Correlations with a coefficient of |r| > 0.8 and *p* < 0.05 were selected, and these relationships were visualized in Figure 5. It is intriguing that phytosphingosine was found to be correlated with DEGs in all three comparison groups (Figure 5A–C). In both the BH vs. HN and YH vs. HN comparisons, phytosphingosine showed a negative correlation with *GPX1*. Conversely, in the LH vs. HN comparison, phytosphingosine was positively correlated with *FHL1*.

Notably, both the BH vs. HN and LH vs. HN comparisons exhibited a negative correlation between *FHL1* and heptanoylcarnitine, with a positive correlation between BTG2 and heptanoylcarnitine. Subsequently, we screened the common metabolic-related pathways annotated by DAMs and DEGs in three comparison groups. Significantly, in the BH vs. HN group, the sphingolipid signaling pathway and proximal tubule bicarbonate reclamation were co-enriched in two comparisons (Figure 5D). Between the LH and HN groups, protein digestion and absorption, GABAergic synapse, phenylalanine metabolism, Chagas disease, and amoebiasis were commonly enriched (Figure 5E). Nonetheless, no shared enriched pathways were identified between the YH and HN groups.

## 4. Discussion

The HN pig is a regional breed from Henan province in China, renowned for its early sexual maturity, strong adaptability, and excellent meat quality. Nonetheless, indigenous breeds like the HN pig demonstrate challenges like a slow growth rate and a low lean meat percentage [24]. Therefore, this study aimed to utilize heterosis to improve HN pigs by crossbreeding with Berkshire, Landrace, and Yorkshire pigs. The present research compared the carcass and muscle characteristics of HN pigs with their hybrid offspring BH, LH, and YH pigs, and for the first time examined potential regulatory mechanisms using transcriptomics and metabolomics. To improve the reliability of comparisons between the hybrid offspring and the HN pigs, pigs with similar body weights and ages were employed to lessen the confounding effects of age and developmental differences. This approach enabled a more accurate evaluation of variations in muscle characteristics, while the combined analysis of transcriptomics and metabolomics enhanced the identification of gene-metabolite interactions and their regulatory pathways.

Carcass and muscle characteristics, including eye muscle area, backfat thickness, and IMF, directly reflect production efficiency and the economic viability of pig farming [25]. The lean meat percentage of lean-type pigs, such as Yorkshire pigs, may surpass 60%, whereas certain local pig breeds often have a lean meat percentage of about 40% [26,27,28]. Hybrid commercial pigs, produced by crossbreeding lean-type pigs with local breeds, often exhibit a lean meat percentages of about 55% [29,30]. In this study, LH and YH pigs of the same age exhibited lean meat percentage of 56.871 ± 1.407% and 57.522 ± 2.613%, respectively. These values were markedly elevated compared to those of HN pigs, which were 45.903 ± 1.397%. This suggests that crossbreeding of HN pigs with Yorkshire and Landrace pigs may significantly enhance the lean meat production capacity of HN pigs, integrating traits from both native and hybrid breeds.

The comparison of carcass and muscle characteristics among the YH, BH, LH, and HN pigs reveals distinct phenotypic advantages associated with each genetic background. The YH group exhibited significantly larger eye muscle area (41.267 ± 2.022 cm^2^) and higher lean meat percentage (57.522 ± 2.613%) compared to the HN group, indicating superior muscle growth and higher lean meat yield. The IMF content of the YH group was 3.167 ± 1.068%. Numerous studies indicate that a small elevation in pig IMF, specifically between 2.5% and 3.5%, promotes flavor and juiciness while also improving consumer acceptability [3,31,32,33]. This suggests that the YH group may provide a balanced advantage by combining high lean meat production efficiency with potentially favorable sensory qualities. In contrast, among the hybrids, the LH group had the highest IMF content (5.033 ± 0.698%) and a comparatively smaller muscle fiber area (3523.835 ± 106.220 μm^2^), traits previously linked to improved flavor and tenderness in previous studies [3,34]. Although increased IMF often enhances meat tenderness, levels exceeding 3.5% may negatively influence consumer perceptions of pork appearance, texture, and aroma, thereby reducing production efficiency [32]. While LH may provide flavour advantages through smaller muscle fibres and increased IMF content, these benefits are often counterbalanced by decreased lean meat yield and produce only limited sensory improvements.

In recent years, RNA-seq technology has been widely used to study the relationship between genes and important economic traits in pigs [35]. To investigate the underlying causes of phenotypic differences between HN pigs and hybrid pigs, this study conducted transcriptomic analysis on the LD tissue of HN pigs and hybrid pigs. Across the three comparative analyses, we identified a subset of DEGs implicated in the negative regulation of fat deposition, including *GDF10*, *NR4A1*, and *SLIT2*, all of which were significantly upregulated in the hybrid groups [36,37,38]. *GDF10* is a distinct member of the TGFβ superfamily with a unique structural feature. It has been shown that overexpressing *GDF10* reduces obesity in mice and regulates lipid metabolism [36]. *SLIT2*, a 180-kDa member of the Slit extracellular protein family, is a *PRDM16*-regulated secreted factor derived from beige adipocytes that enhances energy expenditure, improves glucose homeostasis in vivo, and may consequently reduce fat deposition [37]. *NR4A1*, a transcriptional activator involved in glucose and lipid metabolism, promotes the expression of *GATA2* and *p53*, thereby inhibiting adipocyte differentiation and fat accumulation [38]. Our investigation revealed that the expression of the aforementioned three genes was considerably elevated in hybrid pigs compared to HN pigs, potentially enhancing the lean meat percentage of hybrid pigs via regulation of lipid metabolism and adipocyte development.

While the primary objective of this study was to assess the impact of crossbreeding on the Huainan pigs, comparisons among the hybrid groups offer additional insights into the genetic contributions of different sire breeds. GO enrichment analysis revealed that fat cell differentiation (GO:0045444) was significantly enriched in all three comparison groups, suggesting that hybridization affects adipocyte differentiation (Tables S4–S6). Skeletal muscle cell differentiation (GO:0035914) and the transforming growth factor beta receptor signaling pathway (GO:0007179) were exclusively enriched in the YH vs. HN comparison (Table S5). The activation of transforming growth factor beta receptor is associated with the suppression of adipogenesis and may serve as a crucial mechanism for the decrease of IMF in YH pigs [39].

Alterations in muscle metabolism are usually accompanied by differences in the metabolite profile associated with changes in gene expression [40]. Nutrient utilization efficiency is also significantly influenced by the metabolic profile of muscle. For example, skeletal muscle in pigs with higher feed efficiency (FE) upregulates glycolytic enzymes compared with pigs with lower FE [41]. In this study, the differences in metabolic features and components between hybrid pig groups and HN pigs were investigated using metabolomics. Based on the criteria, the significant differential components were identified, including lipids and lipid-like molecules, organoheterocyclic compounds, benzenoids, and organic acids and derivatives. KEGG pathway analysis was performed to examine the functions of these DAMs. The majority of DAMs in the BH vs. HN comparison were involved in glycerophospholipid metabolism (ko00564), regulation of adipocyte lipolysis (ko04923), and pyruvate metabolism (ko00620) (Figure 4B, Table S11). In contrast, in the comparison of LH and HN, the DAMs were mainly enriched in glycerophospholipid metabolism and pyruvate metabolism. The DAMs in the YH vs. HN comparison exhibited enrichment in pyruvate metabolism. The differences in metabolites at the metabolic level among different groups all involved core energy metabolism pathways (particularly pyruvate metabolism), but the range and type of metabolic changes varied among groups. Pyruvate metabolism, a dynamic process during postnatal growth, is associated with muscle development and energy metabolism [42]. Furthermore, the use of pyruvate may reduce body fat in limit-fed pigs without reducing muscle protein deposition [43]. Consequently, pyruvate metabolism may be a common metabolic pathway shared by the three groups, and its alterations are likely to directly participate in or drive the differences in pig fat deposition and muscle development. In addition, the biosynthesis of unsaturated fatty acids (ko01040) pathway was enriched with the most differential metabolites in the YH vs. HN comparison, indicating differences in lipid metabolic activity between the two groups. Besides structural and energetic effects, unsaturated fatty acids and their derivatives function as signaling molecules, and several studies have shown that this pathway is significantly associated with several studies indicating a substantial correlation with porcine intramuscular and subcutaneous fat [44,45,46]. These factors may collectively account for the observed variations in IMF between YH and HN pigs.

The Pearson correlation analysis indicated a potential regulatory relationship between DAMs and DEGs. Phytosphingosine was mostly linked to DEGs throughout the three groups, indicating its potential role in the regulation of lipid metabolism under different conditions. Phytosphingosine is a long-chain base that constitutes sphingolipid components and is present in certain tissues, including the epidermis and small intestine in mammals [47]. Research indicates that phytosphingosine is crucial for the development of the epidermal permeability barrier and is involved in signal transduction, apoptosis, and stress responses in mammalian cells [48,49,50]. These cellular processes may influence muscle cell function and thereby indirectly affect meat quality traits, including tenderness and water-holding capacity [49]. The robust association between these metabolites and genes offers potential candidates for subsequent functional validation and mechanistic investigations.

In general, this study explains the differences in carcass and muscle improvement of Huainan pigs resulting from crossbreeding with different paternal breeds, employing a combined analysis of transcriptome and metabolome. This research offers a theoretical foundation for further investigations into Huainan pigs and the utilization of local Chinese pig breeds to develop high-quality meat pig strains and novel varieties. The restricted sample size and limited biological replicates in the transcriptomic and metabolomic analysis may reduce the statistical power of our investigation. Consequently, more research using bigger sample sizes and independent validation will be necessary to substantiate these conclusions.

## 5. Conclusions

This study assessed Huainan pigs and three hybrid combinations (BH, LH, and YH) regarding carcass and muscle characteristics, and further conducted transcriptomic and metabolomic analyses. Analysis of muscle characteristics indicated that crossbreeding Huainan pigs with Landrace and Yorkshire pigs resulted in a significant reduction in lean meat yield (*p* < 0.01). The YH group demonstrated a superior lean meat yield while also retaining potential sensory quality benefits. Conversely, while the LH group exhibited advantageous flavour attributes due to a smaller muscle fibre area and higher IMF content, these advantages were limited by decreased lean meat yield and only slight enhancements in sensory qualities. Transcriptomic analysis revealed that *GDF10*, *NR4A1*, and *SLIT2* were significantly upregulated in all three hybrid groups. Additionally, the transcriptomic analysis revealed that the YH pigs exhibited the lowest IMF levels among the crossbred groups, which is consistent with the disparities in pathway enrichment that were identified. Additional metabolomics demonstrated that the composition of metabolites in hybrid and HN pigs differed only slightly. Pyruvate metabolism was the sole pathway that was consistently shared across all three comparisons among the enriched pathways. This suggests that pyruvate metabolism may represent a common metabolic feature in the hybrid groups.

## Figures and Tables

**Table 1:** Composition and nutrient levels of the basal diet (air-dried).

Ingredients	Contents	Nutrient Components	Contents
Corn	58.00	CP	13.13
Soybean meal	14.00	DE/(MJ/kg)	11.33
Wheat bran	9.00		
Grass meal	15.00		
Premix	4.00		
Total	100.00		

**Table 2:** Comparison of carcass traits and muscle characteristics for three hybrid combinations and Huainan pigs.

Traits	HN (*n* = 3)	BH (*n* = 3)	LH (*n* = 3)	YH (*n* = 3)	*p*-Value
Eye muscle area (cm^2^)	31.933 ^b^ ± 1.608	39.333 ^a^ ± 1.167	37.833 ^a^ ± 1.167	41.267 ^a^ ± 2.022	0.013
Lean meat percentage (%)	45.903 ^B^ ± 1.397	50.537 ^AB^ ± 1.047	56.871 ^A^ ± 1.407	57.522 ^A^ ± 2.613	0.007
IMF content (%)	7.653 ^a^ ± 0.251	4.800 ^ab^ ± 0.916	5.033 ^ab^ ± 0.698	3.167 ^b^ ± 1.068	0.025
Muscle fiber area (μm^2^)	4486.000 ^A^ ± 95.970	3788.250 ^BC^ ± 92.813	3523.835 ^C^ ± 106.220	4011.435 ^B^ ± 74.525	0.001

HN, BH, LH, and YH are abbreviations for Huainan pig, Berkshire × Huainan pig, Landrace × Huainan pig, and Yorkshire × Huainan pig, respectively. Data represent mean ± SEM. Different letters (a, b, and A, B, C) indicate significant differences within the same row among groups (*p* < 0.05 and *p* < 0.01, respectively).

## Data Availability

Data will be provided during review.

## Supplementary Materials

The following supporting information can be downloaded at: https://www.mdpi.com/article/10.3390/ani15213144/s1, Table S1. Primers for real-time quantitative PCR. Table S2. Sample quality control statistics data. Table S3. Genes fpkm expression. Table S4. BH vs. HN.GO_Enrichment. Table S5. LH vs. HN.GO_Enrichment. Table S6. YH vs. HN.GO_Enrichment. Table S7. BH vs. HN.KEGG_Enrichment. Table S8. LH vs. HN.KEGG_Enrichment. Table S9. YH vs. HN.KEGG_Enrichment. Table S10. Quantitative results of metabolites. Table S11. BH vs. HN.KEGG_Enrichment. Table S12. LH vs. HN.KEGG_Enrichment. Table S13. YH vs. HN.KEGG_Enrichment.
