# Supplementary material for: RNA Sequencing and Metabolomic Analyses Reveal Differences in Muscle Characteristics and Metabolic Profiles Between Purebred and Crossbred Huainan Pigs
Source: Animals (Basel). 2025 Oct 29;15(21):3144. doi: 10.3390/ani15213144 (PMC12610133; doi:10.3390/ani15213144)
Supplement: Supplementary file 1 [file animals-15-03144-s001.zip › Supplemental Tables S1,S2.pdf]

**Table S1.** Primers for real-time quantitative PCR

| Genes | Gene Bank | Sequence (5′-3′) | Size (bp) |
|---|---|---|---|
| MYL2 | XM_005670649.3 | F: TGGAGCCAATTCCAACGTGT<br>R: AGCAGCAAAAGTGTCCCTCA | 133 |
| MYH7 | NM_213855.2 | F: AGTGTACAACGCTGAGGTGG<br>R: CAGCATGTACTGGTAGGCGT | 106 |
| TNNC1 | NM_001130243.1 | F: GTGGCACAGTGGACTTCGAT<br>R: GCGGAAGAGGTCAGAAAGCT | 101 |
| FHL1 | XM_005657915.2 | F: TGCTCTAAGAAGCTGGCTGG<br>R: CATCCAGCACACTTCTTGGC | 101 |
| PDLIM3 | XM_005671717.3 | F: TGCAGTACTCCGTCCGGTAT<br>R: GGGCCATCTTAGCAGCAACT | 112 |
| DDIT4L | XM_013979005.2 | F: GCTAGACCGTAGCTTCCACC<br>R: CTCTGGCAAGTCGTCTCCTC | 114 |
| GAPDH | XM_021091114.1 | F: CACTGAGGACCAGGTACTGA<br>R: CACCCTACCAGGAAATGAGC | 203 |

**Table S2.** Sample quality control statistics data

| Sample | Raw Data Reads | Valid Data Reads | Valid Ratio (%) | Q20 (%) | Q30 (%) | GC (%) |
|---|---|---|---|---|---|---|
| HN1 | 40735106 | 39433264 | 96.8 | 99.75 | 97.51 | 51.50 |
| HN2 | 42630086 | 41304788 | 96.89 | 99.75 | 97.51 | 51.50 |
| HN3 | 35011052 | 33864992 | 96.73 | 99.75 | 97.47 | 52.00 |
| BH1 | 39784590 | 38538642 | 96.87 | 99.74 | 97.43 | 52.50 |
| BH2 | 35002964 | 33978022 | 97.07 | 99.75 | 97.45 | 51.50 |
| BH3 | 44344944 | 42817002 | 96.55 | 99.75 | 97.56 | 51.50 |
| LH1 | 37048366 | 35923484 | 96.96 | 99.74 | 97.59 | 52.50 |
| LH2 | 35792766 | 34755400 | 97.10 | 99.75 | 97.44 | 52.50 |
| LH3 | 34909600 | 33818910 | 96.88 | 99.74 | 97.43 | 52.50 |
| YH1 | 36871594 | 35621640 | 96.61 | 99.77 | 97.54 | 52.50 |
| YH2 | 37915818 | 36711596 | 96.82 | 99.73 | 97.43 | 52.50 |
| YH3 | 34690370 | 33479402 | 96.51 | 99.74 | 97.44 | 52.00 |
